# Applicability of Transthoracic Contrast Echocardiography for the Diagnosis and Treatment of Idiopathic Pulmonary Arteriovenous Malformations

**DOI:** 10.3389/fcvm.2021.656702

**Published:** 2021-07-12

**Authors:** Yujiao Deng, Xin Huang, Guangyi Wang, Jian Cao, Shengshu Wang, Yue Li, Yiru Wang, Jing Ye, Peifang Zhang, Xiaotian Chen, Yukun Luo, Kunlun He

**Affiliations:** ^1^Department of Ultrasound, The First Medical Center of Chinese PLA General Hospital, Beijing, China; ^2^Department of Cardiology, The Second Medical Center & National Clinical Research Center for Geriatric Diseases, Chinese PLA General Hospital, Beijing, China; ^3^Department of Cardiology, The First Medical Center of Chinese PLA General Hospital, Beijing, China; ^4^Beijing Key Laboratory of Aging and Geriatrics, National Clinical Research Center for Geriatrics Diseases, Second Medical Center of Chinese PLA General Hospital, Institute of Geriatrics, Beijing, China; ^5^Biomind Inc, Beijing, China; ^6^Translational Medicine Research Center, Medical Artificial Intelligence Research Center, Chinese PLA General Hospital, Beijing, China

**Keywords:** pulmonary arteriovenous malformations, idiopathic, right-to-left shunt, embolotheragy, transthoracic contrast echocardiography

## Abstract

**Objective:** To explore the preferred test to screen for pulmonary arteriovenous malformations (PAVMs) and to predict the probability of interventional embolization.

**Methods:** We performed a retrospective observational study evaluating patients with idiopathic PAVMs from 2009 to 2019. After clinical evaluation, a total of 105 patients were studied, including 71 patients with positive digital subtraction pulmonary angiography (DSPA) findings and 34 with negative DSPA findings. The following patient data were assessed: blood test, chest radiograph, transthoracic contrast echocardiography (TTCE), and DSPA findings.

**Results:** The majority of patients with idiopathic PAVMs were female (66.2% with positive DSPA findings). We found a good κ-coefficient of 0.77 with strong consistency for inter observer agreement concerning the pulmonary right-to-left shunt (RLS) grade on TTCE, which was superior to conventional chest radiographs. The positive predictive value (PPV) of the radiographic features for PAVMs on DSPA was 0.83 (95% CI 0.64–1.0) and 0.44 for the possibility of embolization (95% CI 0.19–0.70). The PPV of the shunt grade of PAVMs on DSPA was 0.14 (95% CI 0.01–0.29) for grade 1, 0.74 (95% CI 0.60–0.88) for grade 2, and 0.97 (95% CI 0.92–1.0) for grade 3. The PPVs of pulmonary shunt grades 2 and 3 on TTCE for the possibility of embolization for PAVMs were 0.21 (95% CI, 0.05–0.36) and 0.87 (95% CI, 0.79–0.99), respectively.

**Conclusion:** TTCE is the preferred screening test for PAVMs. The pulmonary RLS grade on TTCE not only identifies the likelihood of PAVMs but also predicts the probability for embolization.

## Introduction

Pulmonary arteriovenous malformations (PAVMs) are abnormal communications between pulmonary arteries and veins, resulting in a right-to-left shunt (RLS) of a different magnitude. A pulmonary RLS may cause some clinical symptoms and complications (such as hypoxemia, stroke, and brain abscess), mostly associated with unprocessed pulmonary arterial blood entering the systemic circulation or paradoxical embolism. PAVMs are rare, occurring in an estimated 38/100,000 individuals according to limited prevalence data (1).

One of the most common causes of PAVMs is hereditary hemorrhagic telangiectasia (HHT), an autosomal dominant disorder, which is also known as Osler-Weber-Rendu syndrome. The clinical diagnosis of HHT is based on the Curaçao Criteria (2), including the presence of spontaneous, recurrent epistaxis, multiple mucocutaneous telangiectases at characteristic sites, visceral AVMs, and a first-degree relative with HHT. The other known causes include hepatopulmonary syndrome (HPS), trauma, actinomycosis, penetrating chest trauma or congenital heart surgery, carcinoma, and Fanconi's syndrome, and so on. The remaining PAVMs with an unclear etiology are classified as idiopathic, without any other signs of HHT or known causes mentioned above. Previous studies show that idiopathic PAVMs are anatomically similar to HHT-related PAVMs (3, 4). While the former is a greater number of solitary PAVMs, the latter is characterized by arteriovenous malformations or telangiectasias in multiple organs. In Western countries, around 60–90% of PAVMs patients are associated with HHT (5, 6). While, a study by Kim et al. suggests that PAVMs are less associated with HHT in Koreans than in Westerners, only about 13.3% (7). And in Japan, Shioya et al. has showed that approximately 15.3% of PAVMs were related to HHT (8). This may be attributed to racial differences, the association of PAVMs with HHT in Asian patients is lower than it is in Western patients. As a study in an Asian country, we decide to invest more effort in PAVMs with non-HHT, especially idiopathic PAVMs.

The goal of this study is to analyze retrospectively the characteristics of idiopathic PAVMs in our single-center in the past 10 years, and to investigate the applicability of transthoracic contrast echocardiography (TTCE) for evaluating idiopathic PAVMs.

## Methods

### Study Population

**Figure 1:**
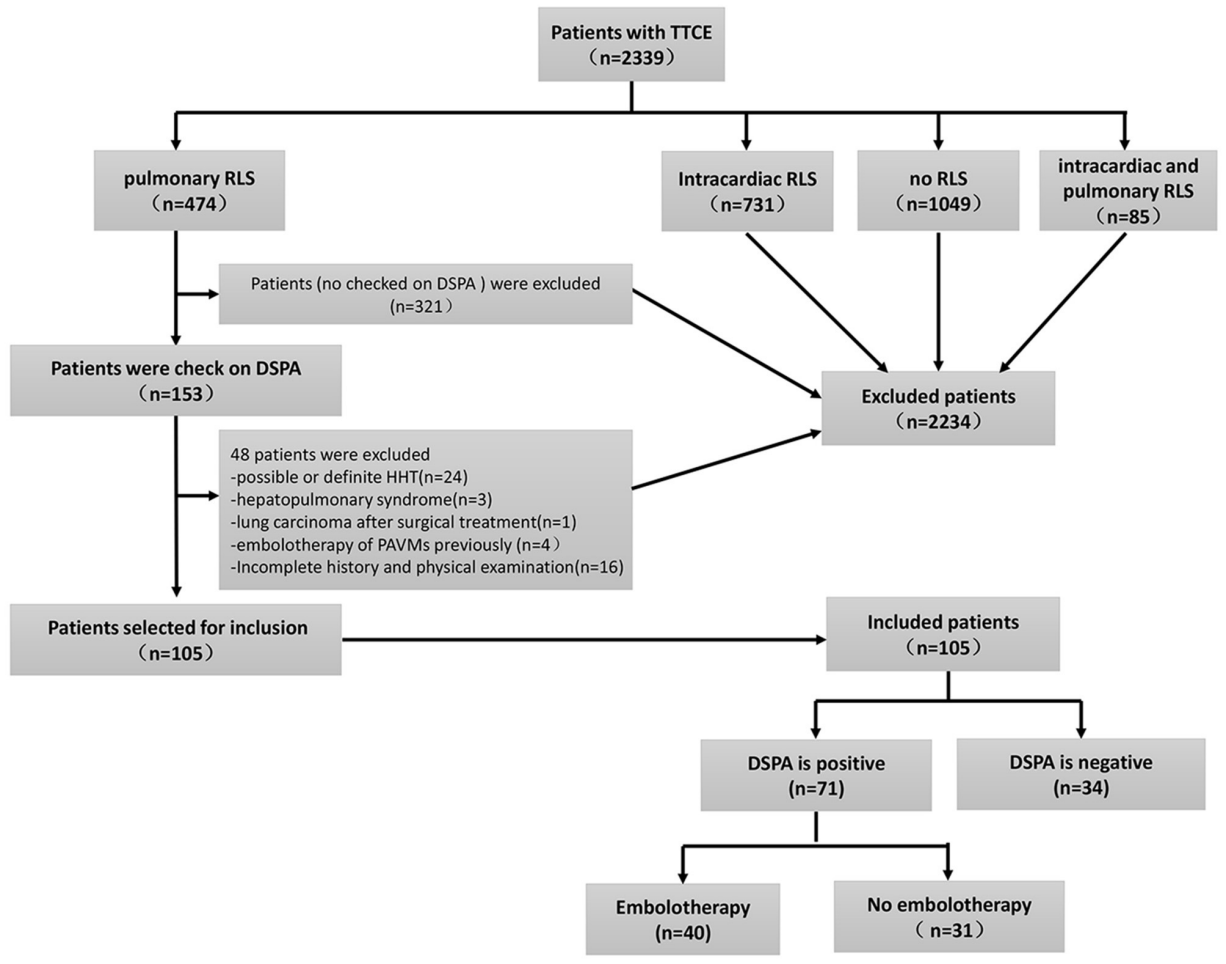
The inclusion flowchart and group assignment of PAVMs patients. Other causes with insufficient clinical information (*n* = 16, 13.5%). TTCE, transthoracic contrast echocardiography; RLS, right to left shuts; DSPA, digital subtraction pulmonary angiography.

We conducted a retrospective observational study with data obtained from electronic medical records to review information from patients with idiopathic PAVMs at the First Medical Center of the PLA General Hospital, China, from January 8, 2009, to December 20, 2019. A total of 2,339 individuals who underwent TTCE were retrospectively enrolled (Figure 1). A total of 105 patients were included in the study, and 71 of them were diagnosed with idiopathic PAVMs (based on DSPA and clinical data); the others had negative DSPA findings. The inclusion criteria were as follows: (1) patients who underwent TTCE; (2) patients with only a pulmonary RLS; (3) patients who underwent DSPA; and (4) patients without a definitive or suspected diagnosis of HHT according to the Curaçao criteria (2) or other known causes; (5) All included patients were made an accurate diagnosis by professionals. These patients came from 16 provinces, cities, and autonomous regions in China. Of the patients with idiopathic PAVMs, 40 patients received embolization, and 31 did not undergo embolization. We assessed the following data from these patients: family history, clinical symptoms, blood tests, abdominal ultrasonography (to exclude arteriovenous malformations in the abdominal organs), transthoracic echocardiography, chest radiographs, TTCE, and DSPA. Fifty-three patients underwent chest CT scans, and 57 patients with suspected stroke underwent cranial CT or MRI to rule out cerebral vessel malformation in our hospital. Fifty-two patients received chest CT scans and 19 patients with cranial CT or MRI in other 3A hospitals. The study was approved by the Institutional Review Board of the First Medical Center at PLA General Hospital.

#### Chest Radiography

Routine chest radiography was performed on all included patients. The classic roentgenographic sign of abnormal cartilage was a round or oval sharply defined mass of uniform density, often lobulated. As the shadow may be hidden by hemothorax, the presence of pleural effusion was assessed. Two experienced radiologists (with dedicated expertise in PAVMs) who were blinded to each patient's medical history evaluated the chest radiographs. In cases of disagreement, the radiographs were re-evaluated by a third radiologist to confirm the final result.

#### Transthoracic Echocradiography

All patients underwent transthoracic echocardiography with a detailed evaluation. The main outcome measures were as follows: left atrial diameter (LAD), left ventricular end-diastolic dimension (LVEDV), right ventricular dimension (RVD), right atrial dimension (RAD), interventricular septal thickness (IVST), left ventricular posterior wall thickness (LVPWT), and left ventricular ejection fraction (LVEF).

#### TTCE

**Figure 2:**
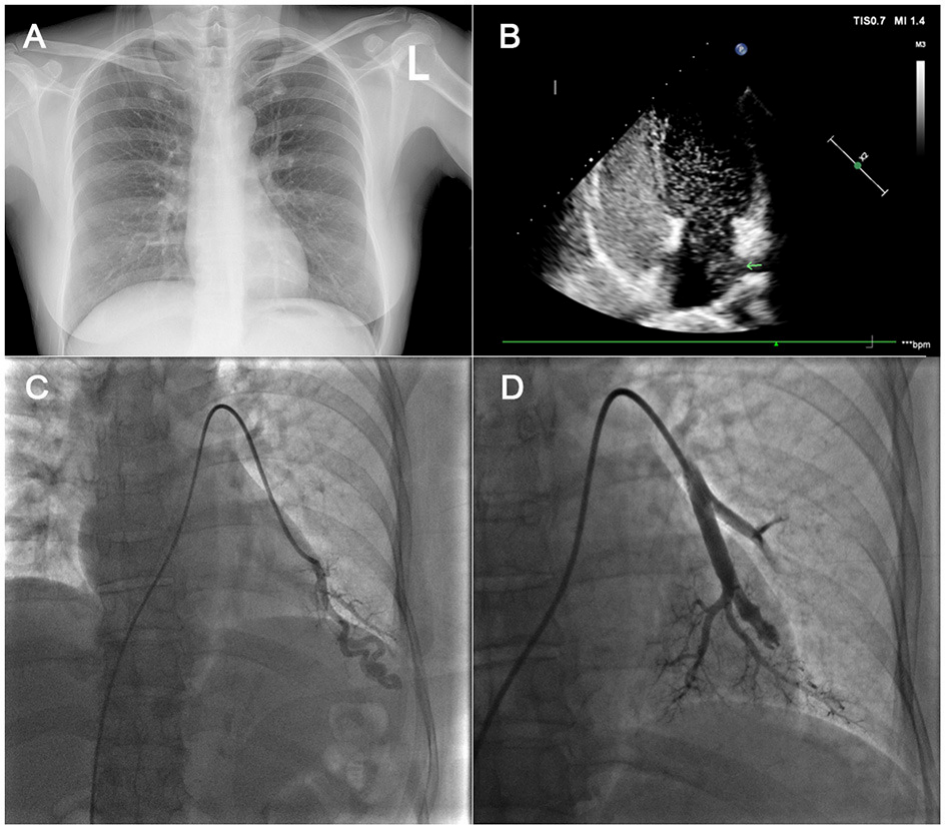
In **(A)**: this patient had a chest X-ray with no abnormalities. In **(B)**, the four-chamber image of CTTE showed markedly positive result and left chamber microbubbles came from the left lower pulmonary vein. In **(C)**, a left lower lobe PAVM was noted on pre-treatment DSPA image. In **(D)**, the PAVM was embolized. TTCE, transthoracic contrast echocardiography; DSPA, digital subtraction pulmonary angiography; PAVM, pulmonary arteriovenous malformation.

**Figure 3:**
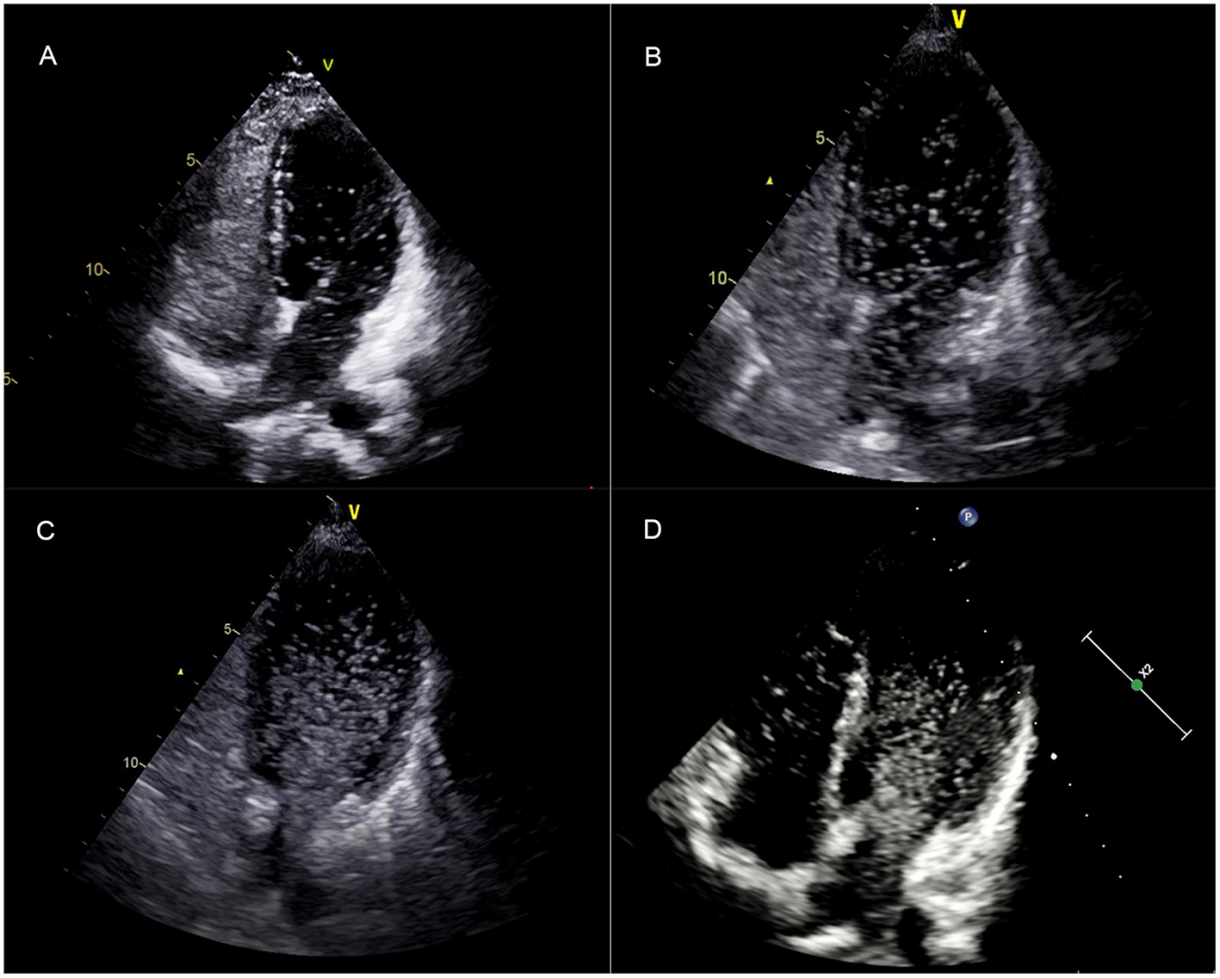
After complete opacification of the right atrium, the TTTE showed the microbubbles in the left chamber after five cardiac cycles in a single frame image. **(A–C)** correspond to a mild (grade 1), moderate (grade 2), and extensive (grade 3) pulmonary RLS, respectively. **(D)** showed that the left chamber microbubbles lasted until the right chamber microbubbles disappeared. TTCE, transthoracic contrast echocardiography; RLS, right to left shunt.

TTCE was performed by experienced echocardiographers and assistants. The assistants prepared two 10-ml syringes connected with a three-way pipe; one was filled with 9 ml of physiological saline solution, and the other with 1 ml of air; these were agitated 20 times in order to create saline contrast (microbubbles). Following the directions of the echocardiographers, saline contrast was administered through an antecubital vein by bolus injection while evaluating the four-chamber apical view. The evaluation method was as follows (9, 10): the potential appearance of microbubbles, its timing, and its hunt origin were permitted to be intuitively observed. The presence of microbubbles derived from the pulmonary veins was classified as a pulmonary RLS (Figure 2). After complete opacification of the right atrium, microbubbles came in a delay of five cardiac cycles, even lasted until right chamber microbubbles disappeared, and was considered positive for pulmonary RLS. Microbubbles entered into the left chamber though patent foramen ovale (PFO) or atrial septal defect within three cardiac cycles, as for intra-cardiac RLS. In the case of RLS uncertainty, transesophageal echocardiography was used to identify whether or not intra-cardiac RLS. The absence of microbubbles in the left chamber in every single frame was considered to indicate the absence of an RLS. We adopted the following standards for the pulmonary RLS grade: based on the maximum number of microbubbles in the left chamber in one still frame, the shunt was graded as 1 (<30 microbubbles), 2 (30–100 microbubbles), or 3 (>100 microbubbles), meaning that the grade 4 shunt described by Barzilai *et al*. is included in our grade 3 pulmonary shunt (11, 12) (Figure 3). Each patient's TTCE grade was determined by two experienced echocardiographers. In cases of disagreement, TTCE views were evaluated again by the third cardiologist to reach a consensus on the final determination.

### DSPA and Treatment Programs

DSPA was performed by interventional cardiologists. Patients who were highly suspected or diagnosed with PAVMs by chest CT underwent DSPA. Following femoral venous access is obtained, weight-adjusted unfractionated heparin (100 u/kg) is given intravenously. Routine right heart catheterization is performed to assess the pulmonary artery pressure. A 6-F directional flush catheter was used to perform biplane angiogram in anteroposterior and ipsilateral 40° oblique projections. Iopromide Injection (Ultravist 370) was injected at a rate of 25 ml/sec for 1 s in the main left or right pulmonary artery. Once identified the position of the PAVMs, selective catheterization of the branch pulmonary artery was performed with a visceral catheter to identify the size and number of the “feeding vessels” of the PAVMs targeted for closure. Selective catheterization of the feeding vessel was performed and hand injection of contrast was used to confirm position and define exact anatomy of the PAVMs to determine site of implantation as well as size of the device to be used. Imaging of the PAVMs was performed by placing a directional coaxial diagnostic catheter system in the feeding artery of the PAVMs and performing hand injection with filming at variable rates, usually four frames per second.

After selective catheterization of the feeding artery, the PAVMs were treated with embolization as close as possible to the sac of the PAVM with Amplatzer devices and/or coils. According to the 2011 HHT consensus guidelines and further researches (13–15), PAVMs with feeding artery diameter 2 mm or larger, and any symptomatic PAVMs were reasonable to perform embolization. And, when an embolization procedure is being performed, all visible PAVMs (such as those smaller than 2 mm) were considered occluded at the same session, if technically feasible.

### Statistical Analysis

Descriptive statistics were used to report patient characteristics. Normally distributed continuous variables are presented as the mean ± standard deviation (SD). Non normally distributed variables are presented as medians with ranges. The positive predictive value (PPV) of each grade on TTCE and characteristic findings on chest radiographs were calculated using DSPA as a reference. The rank-sum test and Ridit analysis were used to determine whether there was a significant association between the echocardiographic shunt grade and the presence of PAVMs on DSPA. Statistical analyses were performed using a statistical software package (SPSS, Version 26.0. Armonk, NY: IBM Corp).

## Results

### Baseline Patient Information

Seventy-one patients (mean age, 36.2 ± 11.8 years, 66.2% females) were diagnosed with idiopathic PAVMs, and 34 patients (mean age, 32.9 ± 12.6 years, 55.9% females) did not have PAVMs identified on DSPA. Their basic information and clinical symptoms are shown in Tables 1, 2. In patients with idiopathic PAVMs, the oxygen saturation of hemoglobin (SpO_2_) levels declined significantly compared with those in patients without PAVMs (94.3 ± 60.5 vs. 98.6 ± 10.2, *p* < 0.001) in Table 2. However, there were no significant differences in the other routine blood and echocardiographic parameters between the two groups. The echocardiographic parameters including LAD, LVEDV, RVD, RAD, IVST, LVPWT, and LVEF were within the normal range.

**Table 1:** The basic information and clinical symptoms of included patients.

**Clinical symptoms**	**Idiopathic PAVMs (*n*, %)**	**No PAVMs on DSPA (*n*, %)**	**Total (*n*, %)**
Patients, *n*	71	34	105
Male	24 (33.8)	15 (44.1)	39 (37.1)
Female	47 (66.2)	19 (55.9)	66 (62.9)
Migraine	29 (40.8)	16 (47.1)	45 (42.9)
Activity chest tightness	26 (36.6)	8 (23.5)	34 (32.3)
Activity shortness of breath	19 (26.8)	5 (14.7)	24 (22.9)
Activity Dizzy	21 (29.6)	11 (32.4)	32 (30.1)
Weak	9 (12.6)	1 (2.9)	10 (8.6)
Cryptogenic stroke	7 (9.9)	–	7 (6.7)
Hemoptysis	5 (7.1)	–	5 (4.8)
Hemothorax	2 (2.8)	–	2 (1.9)
Cyanosis	3 (4.2)	–	3 (2.9)
Cerebral abscesses	2 (2.8)	–	2 (1.9)
Epilepsy	6 (8.1)	–	6 (5.7)
Secondary polycythemia	5 (7.0)	–	5 (4.7)

*DSPA, digital subtraction pulmonary angiography*.

**Table 2:** The clinical data and echocardiography data of included patients.

	**Idiopathic PAVMs**	**No PAVMs on DSPA**	***p***
*N*	71	34	
AGE	36.2 ± 11.8	32.9 ± 2.6	0.186
SBP	119.1 ± 14.2	117.3 ± 13.8	0.556
DBP	73.4 ± 11.2	71.2 ± 9.6	0.320
HGB	139.3 ± 24.5	136.2 ± 14.2	0.515
RBC	4.6 ± 0.8	4.5 ± 0.5	0.403
HCT	0.4 ± 0.1	0.40 ± 0.2	0.086
MCVU	88.7 ± 4.7	88.0 ± 4.8	0.490
MCH	30.2 ± 2.2	30.1 ± 1.9	0.777
RDW	13.6 ± 2.9	12.7 ± 1.5	0.105
AoD	26.6 ± 3.4	27.0 ± 4.2	0.571
PAD	20.3 ± 2.9	21.2 ± 2.8	0.134
RAD	32.2 ± 6.1	30.3 ± 5.6	0.113
RVD	32.5 ± 4.9	31.5 ± 3.7	0.295
LAD	29.8 ± 4.2	29.8 ± 3.7	0.978
LVEDD	45.1 ± 3.3	44.2 ± 3.7	0.822
IVST	9.0 ± 1.2	9.1 ± 1.6	0.833
LVPWT	8.8 ± 1.3	8.4 ± 1.2	0.150
LVEF	64.0 ± 4.7	64.2 ± 4.6	0.843
SpO_2_	94.3 ± 6.5	98.6 ± 1.2	<0.001

*HBG, hemoglobin; RBC, red blood cell count; HCT, hematocrit; MCVU, mean corpuscular volume; MCH, mean corpuscular hemoglobin; RDW, red cell distribution width; AoD, Aortic Dimension;PAD, pulmonary artery internal dimension; RAD, right atrial dimension; RVD, right ventricular dimension; LAD, left atrial diameter; LVEDV, left ventricular diastolic volume; IVST, interventricular septum thickness; LVPWT, left ventricular posterior wall thickness; LVEF, left ventricular jection fraction; SpO_2_, saturation of hemoglobin with oxygen; DSPA, digital subtraction pulmonary angiography*.

#### Chest Radiography

The κ-coefficient was 0.57 with reasonable consistency for inter observer agreement concerning PAVMs identified on chest radiographs. The accuracy, sensitivity, false-positive rate, false-negative rate of chest radiographs for detecting PAVMs were 43.8%, 21.1%, 8.8%, and 78.9%, respectively. The PPV of the presence of characteristic radiographic features was 0.83 (95% CI, 0.64–1.0) for the detection of PAVMs on DSPA and 0.44 for the possibility of embolization (95% CI, 0.19–0.70). The PPV of negative chest radiographic findings was 0.36 (95% CI, 0.25–0.46) for the presence of PAVMs on DSPA and 0.37 for the possibility of embolization (95% CI, 0.26–0.47) (Table 3).

**Table 3:** Number of patients and PPV for Chest-X results and TTCE grade.

	**Chest-X (+)**	**Chest-X (–)**	**TTCE 1**	**TTCE 2**	**TTCE 3**
Number	18	87	27	39	39
PPV^※^	0.83	0.36	0.14	0.74	0.97
95%CI^※^	0.64–1.0	0.25–0.46	0.01–0.29	0.60–0.88	0.92–1.0
PPV^∞^	0.44	0.37	0	0.21	0.87
95%CI^∞^	0.19–0.70	0.26–0.47	–	0.05–0.36	0.79–0.99

*PPV: positive predictive value; CI: confidence interval; TTCE: transthoracic contrast echocardiography*;

※ *:whether presence of PAVMs on DSPA (p < 0.0001 for trend)*.

∞ *:whether embolization for positive DSPA patients (p < 0.0001 for trend)*.

#### TTCE

TTCE documented a grade 1 pulmonary RLS in 27 (25.8%) patients, grade 2 in 39 (37.1%) patients, and grade 3 in 39 (37.1%) patients (Table 4). We found a good κ-coefficient of 0.77 with strong consistency for inter observer agreement concerning the pulmonary RLS grade on TTCE. The PPV of the shunt grade measurement for the presence of PAVMs on DSPA was 0.14 (95% CI, 0.01–0.29) for grade 1, 0.74 (95% CI, 0.60–0.88) for grade 2, and 0.97 (95% CI, 0.92–1.0) for grade 3. The PPVs of pulmonary shunt grades 2 and 3 on TTCE for the possibility of embolization of PAVMs were 0.21 (95% CI, 0.05–0.36) and 0.87 (95% CI, 0.79–0.99), respectively (Table 3).

**Table 4:** DSPA results and treatments in different TTCE grade.

**TTCE Grade**	**DSPA**		**Total**	**Treatment (*****n*** **= 105)**			**Total**
	**+**	**–**		**With** **embolotherapy**	**Without** **embolotherapy**	**No related** **treatment**	
1	4	23	27	0	4	23	27
2	29	10	39	6	23	10	39
3	38	1	39	34	4	1	39
Total	71	34	105	40	31	34	105

*TTCE, transthoracic contrast echocardiography; DSPA, digital subtraction pulmonary angiography; +, positive; –, negative*.

#### DSPA

Of the 71 patients with idiopathic PAVMs, 53 patients (74.6%) had solitary PAVMs, and 18 patients (25.4%) had two or more PAVMS identified on DSPA. Furthermore, 98 PAVMs showed flow distribution in the bilateral pulmonary arteries: 91 out of 98 (92.9%) were simple PAVMs, and only seven were diffuse/complex (7.1%). Of all PAVMs, 38 (37.8%) were located in the left lower lobe, and 31 (31.6%) were located in the right lower lobe. Sixty-four PAVMs were embolized with Amplatzer devices and/or coils without any postoperative complications (Table 5). The SpO_2_ levels in patients increased significantly between the preoperative period and postoperative period following embolization (91.87 ± 0.7 vs. 97.03 ± 0.4, *p* < 0.01).

**Table 5:** The locations and proportions of PAVMs by DSPA.

**PAVMs locations**	**Numbers of PAVMs**	**Numbers of embolization**
RUL	9 (9.2%)	5(7.8%)
RML	10 (10.2%)	6(9.4%)
RLL	31 (31.6%)	19 (29.7%)
LUL	12 (12.2%)	8 (12.5%)
LLL	38 (37.8%)	26 (40.6%)
Total	98	64

*PAVMs, pulmonary arteriovenous malformations; RLL, right lower lobe; RML, right median lobe; RUL, right upper lobe; LUL, left upper lobe; LLL, left lower lobe*.

## Discussion

In recent years, some symptoms, such as migraine and cryptogenic stroke, have been suggested to might be partially related to RLS. PFO is widely known to be the most common cause of an intracardiac RLS. Other types of RLS, other than PFO-RLS, mainly originate from the lungs and are called pulmonary RLS (P-RLS), with the incidence rate between 20 and 41% (9). The main cause of pulmonary RLS at the pulmonary level is PAVMs. PAVMs are abnormal capillary developments including capillary dysplasia or the disappearance of the vascular septum of the arteriovenous plexus. Previous studies have shown that PAVMs are associated with disabling and life-threatening complications, such as cryptogenic stroke, cerebral abscesses, and hemothorax. Those studies have mostly focused on HHT-related PAVMs, and the incidence of cerebrovascular accidents and cerebral abscesses has been shown to be 18–35% (16–18). These complications have been found in patients with idiopathic PAVMs, but the incidence rate is lower than that in patients with HHT-related PAVMs. According to our statistical analyses, migraines are relatively common. These results suggest that RLSs may play an important role in the pathogenesis of migraine attacks. Metabolites, such as serotonin, nitric oxide, and kinins can bypass the pulmonary circulation in the presence of an RLS and enter the systemic circulation to irritate the trigeminal nerve and brain vasculature, thereby producing migraines (19). Past researchers have assumed that RLSs were closely associated with PFO, ignoring the link of PAVMs with pulmonary RLSs.

In this study, 71 patients with idiopathic PAVMs showed 98 PAVMs (except for two patients who had bilateral diffuse PAVMs) on DSPA. Idiopathic PAVMs were distributed in all areas of the lungs, and the predominant pattern observed was the bilateral involvement of the inferior lobes of the lungs. This phenomenon was consistent with the findings of other studies (4, 20) and may be a consequence of blood flow redistribution to the site of PAVMs in the upright position (21, 22). PAVMs were mainly composed of three kinds of morphological structures (23, 24): simple, complex, and rare diffuse PAVMs. The vast majority of idiopathic PAVMs (92.9%) were simple, and only a few (7.1%) were diffuse/complex. A notable difference was that idiopathic PAVMs were often solitary (~74.6% in this study), while HHT-related PAVMs were less likely to be solitary (~40% in previous studies) (25, 26). Embolization is suggested for patients with idiopathic PAVMs, and there are no differences between the HHT-PAVMs and idiopathic PAVMs about treatment safety and efficacy (27). Mager et al. (28) stated that patients with single PAVMs were more likely to benefit after embolization than patients with multiple PAVMs. This study showed that patients' SpO_2_ levels increased after embolization without any complication. Chest radiographs and TTCE can be used for PAVM screening. In this study, both diagnostic methods showed good inter observer agreement, but TTCE showed better agreement than chest radiography. The κ-coefficient was 0.57 for chest radiography with classic roentgenographic signs and 0.77 for TTCE with regard to the pulmonary RLS grade. Although the PPV of chest radiographs for diagnosing PAVMs was up to 83.1%, this diagnostic method had low sensitivity, and the false-negative rate was as high as 78.9%. The false negative may be generated by the shadow of the rib or heart. The noise and low contrast of the chest X-ray image made it difficult to recognize the abnormalities. While TTCE was unaffected about it and can be more valuable, including predicting the possibility of PAVMs and whether they need embolization. Based on our results, in the vast majority of patients with a grade 1 pulmonary RLS, no PAVMs were observed; the remaining patients had small PAVMs that did not need embolization. For those patients, chest CT, multi-detector CT pulmonary arteriography (MCTPA), and DSPA were unnecessary because they were unlikely to benefit from these tests. However, follow-up TTCE reexamination of the shunt grade every 5 years is worthwhile (29). Patients with pulmonary RLS grade 2 had a higher proportion of detected PAVMs. However, only a few of these patients required transcatheter embolization. A grade 3 pulmonary RLS was associated with an increased prevalence of PAVMs, with most patients needing embolization. The findings of this study are consistent with those of previous research about PAVMs (30). Thus, TTCE grading enabled the detection of potentially treatable PAVMs, which is of great importance in clinical practice.

DSPA, as the gold standard test for PAVM, is not only for the diagnosis of PAVMs but also is a part of an embolization procedure. Chest CT, Contrast-enhanced magnetic resonance angiography (MRA), and radionuclide perfusion lung scanning are considered to be alternative less invasive methods. Chest CT has played a very important role in PAVMs diagnosis and enables sharp images of feeding arteries, sacs, and draining veins by its high spatial resolution (23). However, ionizing radiation is its drawback which should not be neglected in chest CT. Hanneman et al. reported patients with an average of four chest CT scans (range 0–20) may be at risk for radiation induced harm (31). As technology progresses, low-dose, and ultra-low-dose (sub-millisievert) CT have significantly lowered radiation burden (32). While the “as low as reasonably achievable” principle applies in radiology and MRA offers the possibility of high-quality images of PAVMs without radiation burden (33). Furthermore, chest CT is inferior to DSPA in the analysis of angioarchitecture of PAVMs (26% vs. 60%) (34). MRA can be used to assess PAVMs patency by analyzing arteriovenous enhancement kinetics and it is regarded as a feasible, efficient and promising tool for non-invasive assessment of PAVMs (35). Yet it is worth noting that there are also some weaknesses needed to improve, such as long processing times, big artificial error, etc. (33).

Pulmonary capillaries are the smallest vessels (7–10 μm in diameters and never exceeding 15 μm) and have a distinctive function as biological filters (36). TTCE with agitated saline provides microbubbles with a reported size distribution of 24–144 μm; the majority are 30–70 μm in diameter (37). Microbubbles have difficulty traveling through pulmonary capillaries under normal conditions. PAVMs allow pulmonary blood flow to bypass the pulmonary capillaries; thus, microbubbles can be visualized in the left ventricle. We also noticed a false-positive phenomenon: TTCE showed a grade 1 pulmonary RLS in 85.2% patients, a grade 2 pulmonary RLS in 25.6% patients, and a grade 3 pulmonary RLS in 2.6% patients, but there were no PAVMs detected on DSPA. Some scholars have concluded that TTCE can identify microscopic PAVMs or telangiectasia below the detection limit of DSPA or CT scans (30). Other scholars have suggested that false-positive findings may be associated with the presence of intrapulmonary arteriovenous anastomoses (36). Intrapulmonary arteriovenous anastomoses are vascular conduits ≥50 μm (up to 500 μm) causing a “breach” in the pulmonary circulation that allows pulmonary blood flow to bypass the pulmonary capillaries. This is observed in ~30% of healthy adults at rest and more during hypoxia, exercise, and changes in body position (38). In contrast, PAVMs are pathological, fixed, and constantly allow blood to flow through them. So, there should be no doubt that, the SpO_2_ levels decline obviously in idiopathic PAVMs patients, compared with those in patients without PAVMs (94.36 ± 0.5 vs. 98.61 ± 0.2, *p* < 0.001). Since embolization, arteriovenous deformed channels are interrupted without fixed, and constantly pulmonary RLS, then, the SpO_2_ levels increased significantly compared the preoperative period with postoperative period (91.87 ± 0.7 vs. 97.03 ± 0.4, *p* < 0.01).

## Study Limitations

First, this was a retrospective single-center study based on a small sample of patients with idiopathic PAVMs, and many factors may have led to weakened statistical characteristics. Interestingly, the patients came from 16 provinces, cities, and autonomous regions in China, and there were no family-based linkages. This factor is consistent with the sporadic characteristics of idiopathic PAVMs, and we believe that it significantly contributes to literature. Second, not all the patients had complete genetic data. The diagnosis of PAVMs is currently still a clinical diagnosis, and existing genetic testing methods are only 80% sensitive (39). Three cardiovascular specialists experienced in the diagnosis and treatment of PAVMs conducted this study. Last but not least, for a retrospective study, chest CT images are implemented in our hospital and other 3A hospitals. There may be an inherent selection bias and inconsistency in the different display states. So, we haven't issued a comprehensive analysis about chest CT images.

## Conclusions

Pulmonary RLSs are associated with some symptoms possibly caused by a paradoxical embolism or metabolites that bypass pulmonary circulation; thus, they are worthy of attention. TTCE is preferred for PAVM screening due to its excellent sensitivity, good inter observer reproducibility, non-invasive nature, low risk and costs, and lack of radiation concerns. The pulmonary RLS grade on TTCE not only increases the likelihood of detecting PAVMs but also helps to predict the need for embolization. Idiopathic PAVMs are mostly solitary, and they can benefit from embolization.

## Data Availability Statement

The raw data supporting the conclusions of this article will be made available by the authors, without undue reservation.

## Ethics Statement

The studies involving human participants were reviewed and approved by Chinese PLA General Hospital Medical Ethics Committee. Written informed consent for participation was not required for this study in accordance with the national legislation and the institutional requirements.

## Author Contributions

KH, YL, YD and XH were designed this study and analyzed and interpreted the patient data, drafting the manuscript, and control and guarantee that all aspects of the work was investigated and resolved. GW, JC, SW, YL, YW, JY, PZ, and XC acquisition of data, analysis and interpretation of data, revising the manuscript, and control and guarantee that all aspects of the work was investigated and resolved. All authors read and approved the final manuscript.

## Conflict of Interest

JY, PZ, and XC were employed by the company Biomind Inc. The remaining authors declare that the research was conducted in the absence of any commercial or financial relationships that could be construed as a potential conflict of interest.
